# The Natural Protoalkaloid Methyl-2-Amino-3-Methoxybenzoate (MAM) Alleviates Positive as well as Cognitive Symptoms in Rat and Mouse Schizophrenia Models

**DOI:** 10.2174/1570159X21666230720122354

**Published:** 2023-07-24

**Authors:** Yami Bright, Dorien A. Maas, Michel M.M. Verheij, Maria S. Paladini, Helene I.V. Amatdjais-Groenen, Raffaella Molteni, Marco A. Riva, Gerard J.M. Martens, Judith R. Homberg

**Affiliations:** 1 Department of Cognitive Neuroscience, Donders Institute for Brain, Cognition and Behaviour, Radboud University Medical Centre, Nijmegen, The Netherlands;; 2 Department of Molecular Animal Physiology, Donders Institute for Brain, Cognition and Behaviour, Faculty of Science, Nijmegen, The Netherlands;; 3 Department of Anatomy and Neurosciences, Amsterdam Neuroscience, Amsterdam UMC, Vrije Universiteit Amsterdam, Amsterdam, The Netherlands;; 4 Department of Pharmacological and Biomolecular Sciences, Universita’ degli Studi di Milano, Milan, Italy;; 5 Altos Labs Bay Area Institute of Science, Altos Labs, Inc., Redwood City, CA, USA;; 6 System Chemistry, Institute for Molecules and Materials, Radboud University, Nijmegen, The Netherlands;; 7 Department of Medical Biotechnology and Translational Medicine, Universita’ degli Studi di Milano, Milan, Italy;; 8 Biological Psychiatry Unit, IRCCS Istituto Centro San Giovanni di Dio Fatebenefratelli, Brescia, Italy

**Keywords:** MAM, antipsychotics, schizophrenia, APO-SUS, PPI, gnawing, catalepsy, procognitive

## Abstract

The development of new antipsychotics with pro-cognitive properties and less side effects represents a priority in schizophrenia drug research. In this study, we present for the first time a preclinical exploration of the effects of the promising natural atypical antipsychotic Methyl-2-Amino-3-Methoxybenzoate (MAM), a brain-penetrable protoalkaloid from the seed of the plant *Nigella damascena*. Using animal models related to hyperdopaminergic activity, namely the pharmacogenetic apomorphine (D2/D1 receptor agonist)-susceptible (APO-SUS) rat model and pharmacologically induced mouse and rat models of schizophrenia, we found that MAM reduced gnawing stereotypy and climbing behaviours induced by dopaminergic agents. This predicts antipsychotic activity. In line, MAM antagonized apomorphine-induced c-Fos and NPAS4 mRNA levels in post-mortem brain nucleus accumbens and dorsolateral striatum of APO-SUS rats. Furthermore, phencyclidine (PCP, an NMDA receptor antagonist) and 2,5-Dimethoxy-4-iodoamphetamine (DOI, a 5HT2A/2C receptor agonist) induced prepulse inhibition deficits, reflecting the positive symptoms of schizophrenia, which were rescued by treatment with MAM and atypical antipsychotics alike. Post-mortem brain immunostaining revealed that MAM blocked the strong activation of both PCP- and DOI-induced c-Fos immunoreactivity in a number of cortical areas. Finally, during a 28-day subchronic treatment regime, MAM did not induce weight gain, hyperglycemia, hyperlipidemia or hepato- and nephrotoxic effects, side effects known to be induced by atypical antipsychotics. MAM also did not show any cataleptic effects. In conclusion, its brain penetrability, the apparent absence of preclinical side effects, and its ability to antagonize positive and cognitive symptoms associated with schizophrenia make MAM an exciting new antipsychotic drug that deserves clinical testing.

## 1 INTRODUCTION

Schizophrenia (SZ) is a psychiatric disorder characterized by positive symptoms (psychosis), such as hallucinations and delusions, as well as negative symptoms, such as blunted emotions, social withdrawal, and cognitive dysfunction [1]. SZ treatment relies on both typical (TAPs) or atypical antipsychotic drugs (AAPs). TAPs, such as haloperidol, possess high antagonist potency at dopamine D2 receptors. Unfortunately, D2 antagonism leads to many side effects, including extrapyramidal symptoms (EPS) and hyperprolactinemia [2]. With respect to their mechanism, AAPs have been suggested to be dual antagonists at both D2 and 5HT2A receptors [3].

There is no compromise yet about the definition of “atypicality”. In its original clinical sense, an atypical antipsychotic lacks EPS [4, 5]. However, AAPs have been found to be associated with other major side effects, including weight gain, hyperlipidemia, and type II diabetes [6]. In comparison to TAPs and AAPs, clozapine (CLOZ) has shown superiority in treating individuals with poor symptom response to previous antipsychotics (Aps) and in reducing suicidality in schizoaffective disorders and SZ [7]. However, severe agranulocytosis and other adverse effects of CLOZ limit its more widespread use [8]. As CLOZ is still the best functional Ap, it was used as a control in the present study.

While several efforts have been made to develop Aps that bypass the dopamine system, blockade of the dopamine D2 receptor remains a necessary and sufficient condition for antipsychotic activity [9-12]. For this reason, animals were treated with the D2/D1 receptor agonist apomorphine in dopamine-mediated behavioural experiments. Partial dopamine-D2 receptor agonism with weak but significant positive intrinsic activity has also been used as a strategy to reduce positive symptoms. Drugs with this profile bypass the side effects presented by current AAPs and thought to alleviate the cognitive deficits associated with schizophrenia [13, 14]. Aripiprazole is an example of a dopamine-D2 partial agonist approved by the FDA. However, the compound retained the 5-HT2A and other receptor binding profiles as is common to AAPs and did not show any cognitive advantage over AAPs [12, 15].

Cognitive disability is considered a core symptom of SZ. AAPs produce a slight improvement (if any) in a global cognitive index, but patients continue to experience significant cognitive dysfunction [16]. Because AAPs treat only psychosis and cause serious side effects, we need to move to another approach to treat the various aspects of SZ [17].

Prepulse inhibition (PPI) has been the most extensively investigated feature of SZ and gained widespread interest because it can be assessed in humans and animals with almost identical techniques [18]. Thus, PPI provides a strong translational measure of inhibitory sensorimotor gating mechanisms that are impaired in SZ patients. Similar deficits are produced in rats by pharmacological manipulations [19]. The NMDA receptor antagonist phencyclidine (PCP) and the 5HT2A/2C receptor agonist 2,5-Dimethoxy-4-iodoamphetamine (DOI) induce PPI disruption in animal models, showing sensitivity to only AAPs but not to TAPs. PCP- and DOI-induced PPI deficits represent the most popular and strong animal models used in the preclinical screening for new AAP drugs [19-21].

The APO-SUS rat is a well-characterized pharmacogenetic animal model that displays SZ-relevant features during adulthood, such as altered density of central dopamine receptors [22], high sensitivity to dopaminergic drugs (*i.e*., apomorphine and amphetamine) [23-25], PPI and latent inhibition deficits [26, 27], increased novelty-induced exploration and accumbal dopamine response [23, 28], mPFC parvalbumin interneuron hypomyelination [29] as well as cognitive deficits [29, 30]. The dopamine D2/D1 receptor agonist apomorphine induces high stereotypy scores in APO-SUS rats. In this study, the antagonism of stereotypy was used to assess the antidopaminergic activity of the natural antipsychotic protoalkaloid Methyl-2-Amino-3-Methoxybenzoate (MAM).

MAM was isolated for the first time by Fico *et al.* [31] from the seed essential oil of the plant *Nigella damascena*. Very little is known about this metabolite and only activity against herpes simplex virus type-1 has been found [32]. MAM is a monoamine alkaloid whose antipsychotic activity was predicted by us based on some preliminary theoretical structural pharmacodynamics and preclinical observational studies. Natural drugs are increasingly considered a new therapeutic approach since they contain a large number of compounds with high biological activity [33]. However, there is a widespread misconception that “natural” always means “safe”, as many of these compounds are inherently toxic [34, 35]. Therefore, we explored whether toxicity and other side effects occur with MAM treatments that reach up to 10 times at the most antipsychotic-effective dose of this natural drug.

In neuropsychopharmacology, brain passage is another major challenging aspect besides toxicity. The blood-brain barrier (BBB) excludes ~100% of large-molecule neurotherapeutics and more than 98% of all small-molecule drugs from the brain [36]. Only high lipophilic drugs with a small molecular weight (less than 400 Dalton threshold) are free to cross the BBB. Thus, the time that is needed to develop central nervous system (CNS) drugs is normally much longer than for non-CNS drugs [37]. For this reason, we studied the brain penetrability of MAM after systemic injection. Small molecules with brain penetrability and less toxicity and side effects, especially protoalkaloids like MAM, can provide major pharmacological advantages.

In view of the above, in the present study, we have explored the preclinical brain penetrability of MAM, its antipsychotic-like effects, its cognitive enhancement effects, its possible induction of EPS, and its subchronic toxicity. To this end, we used a combination of powerful rodent models and behavioural tests for SZ related to positive symptoms (apomorphine-induced gnawing stereotypy in APO-SUS rats, apomorphine-induced hyperlocomotion in mice, and DOI-/PCP-induced PPI deficits in Wistar rats) and cognitive symptoms (spatial working memory and reversal learning deficit in APO-SUS rats). The observed absence of serious preclinical side effects, its cognitive enhancement effects and brain penetrability make MAM a promising natural antipsychotic drug with major advantages over existing antipsychotic drugs.

## 2 MATERIALS AND METHODS

### 2.1 Animals

The generation of the APO-SUS and APO-UNSUS rat lines with a high and low susceptibility for apomorphine, respectively, has been described previously by Cools *et al.* [23], Maas *et al*. [29]. Briefly, APO-SUS and APO-UNSUS rat lines have been selectively bred from an outbred Nijmegen Wistar rat population. Wistar rats that displayed stereotyped gnawing behaviour (> 500 gnaws in 45 min) upon injection of the D2/D1 receptor agonist, apomorphine, were selectively bred as APO-SUS rats. The same selective breeding was performed with the rats that showed a weak apomorphine-induced stereotypy (< 10 gnaws in 45 min; APO-UNSUS). Apomorphine injection and behavioural selection were only performed with the first 15 generations of APO-SUS and APO-UNSUS rats. In the subsequent breedings, APO-SUS rats displayed SZ-relevant features without this pharmacological treatment. This rat model has been used for decades as a schizophrenia model. Adult male APO-SUS/UNSUS rats (7 weeks on arrival, Nijmegen, The Netherlands) were used for both the gnawing box test and the T-maze test. Adult male Swiss albino mice (25-30 g) were used in the preliminary experiments related to climbing, catalepsy, and toxicity (Supplementary data (SD)). The animals were drug- and experiment-naive, and all rats and mice were used only once. Upon arrival, the rats were pair-housed in Makrolon type III cages and mice in polycarbonate cages (6 per cage) and kept on a 12:12-h light/dark cycle in a temperature-controlled (25 ± 1°C) colony room. The experimental procedures were approved by the Dutch National Ethics Committee and conducted in accordance with Dutch legislation. Every effort was made to minimize the number of animals used and their suffering.

### 2.2 Drugs

MAM (Pure freebase (> 95%) from ARKPHARM, USA) was used. All dosages are expressed as freebase dissolved in DMSO/Tween/Saline (2.5:2.5:95) and injected subcutaneously in a volume of 5 mL/kg. MAM showed a better effect subcutaneously when compared to the i.p route, at least in the gnawing test. Clozapine hydrochloride and olanzapine hydrochloride (Sigma Aldrich, Germany) were dissolved in saline with some drops of HCl (0.1 M) and pH adjusted to ∼5.2, and injected subcutaneously in a volume of 5 mL/kg. Haloperidol (Janssen-Cilag BV, 50 mg/ml ampule of 1ml, the Netherlands) was diluted in saline (0.9% NaCl in sterile distilled water) to obtain an appropriate dose and given intraperitoneally in a volume of 10 mL/kg. Apomorphine hydrochloride (Duchefa pharm B.V, the Netherlands), PCP (phencyclidine), and DOI (2,5-Dimethoxy-4-iodoamphetamine) (RBI, USA), were dissolved in physiological saline (0.9% NaCl in sterile distilled water) and given subcutaneously in a volume of 5 ml/kg.

### 2.3 Antipsychotic Effect (Antidopaminergic Activity)

#### 2.3.1 Antagonism of Apomorphine-induced Gnawing Stereotypy in APO-SUS Rat Model

The gnawing stereotypy test has been described by Cools *et al*. [23] and modified slightly from Ljungberg and Ungerstedt [38]. APO-SUS rats that have been administered apomorphine started to almost immediately gnaw strongly and reached the highest score of gnawing stereotypy at 30 min. The vehicle itself (DMSO/Tween-80/Saline 2.5% 2.5/ 95%: *v/v*), CLOZ (10 mg/kg) or MAM (3, 10, and 30 mg/kg), were administered subcutaneously 15 minutes before apomorphine was subcutaneously administered at 1.5 mg/kg to the APO-SUS rats. Control groups of both APO-UNSUS/ SUS rats received vehicle + apomorphine and another control group of APO-SUS received vehicle + saline in the same condition. Immediately thereafter, the rats were placed into a gnawing box that contained 32 holes surrounded by concentric ridges to promote stereotypic gnawing behavior. All rats were placed in this box for 45 min, and the gnawing count was automatically recorded every 3 min by an integrated software program.

### 2.4 Atypical Antipsychotic Effect

#### 2.4.1 DOI and PCP-induced Prepulse Inhibition Deficits in Wistar Rats

Startle reactivity was measured as previously described by Ellenbroek *et al*. [26] with slight modifications. The groups of rats tested for DOI and PCP were different and tested only once. Rats were individually housed 3 days before the first PPI session and handled for 5 min/day at least three times. Startle reactivity was measured using two San Diego instruments (San Diego, CA), startle chambers and SR-LAB software. Each chamber had a clear nonrestrictive Plexiglas cylinder (diameter 8.2 cm, length 25 cm) placed in a ventilated and sound-attenuated box. The tube was mounted on a plastic frame, under which a piezoelectric accelerometer was mounted, which recorded and converted the startle into analog signals. A high-frequency loudspeaker inside each chamber produced background noise of 70 dB as well as various acoustic stimuli. Calibration was performed with every cohort to ensure the accuracy of the sound levels and startle measurements. During test sessions, rats were placed into the startle chambers and each testing session began with 5-min acclimatization during which a background noise (70 dB) was presented and remained constant for the entire testing period. After this period, the rats received 10 startle trials, 10 no-stimulus trials, and 40 PPI trials. The intertrial interval was between 10 and 20 seconds with an average of 15 sec presented in a pseudorandom order. The startle trial consisted of a single 120 dB[A] white noise burst lasting 20 ms. The PPI trials consisted of a prepulse (20 ms burst of white noise with intensities of 73, 75, or 80 db[A]) followed, 100 ms later, by a startle stimulus (120 dB[A], 20 ms white noise). Each of the three prepulse trials (73, 75, or 80 dB[A]) was presented 10 times. The no-stimulus trials consisted of background noise only. This represents a control trial for detecting differences in overall activity. Since no such differences were detected, the results are not shown.

#### 2.4.2 Drug Treatments

After 30 min acclimatization in the experimental room, rats were pretreated subcutaneously with the vehicle, CLOZ (10 mg/kg) or MAM (3, 10, 30 mg/kg), 15 min before subcutaneous injection of DOI (3 mg/kg) or PCP (1.5 mg/kg). Rats were placed into the startle chambers 5 min after drug injection (DOI or PCP). Control groups received vehicle prior to saline for both experiments in the same conditions. Both doses of PCP and DOI were selected from prior PPI studies in Wistar rats [39, 40]. The amount of PPI was calculated as a percentage score for each acoustic prepulse intensity:

% PPI = 100− {[(startle response for prepulse + pulse trials)/ (startle response for pulse-alone trials)] ×100}.

The magnitude of the response was calculated as the average responses to all of the pulse-alone or prepulse trials.

### 2.5 Cognitive Enhancement-like Effects

#### 2.5.1 Spatial Working Memory Deficits in APO-SUS Rat (T-maze Test)

##### 2.5.1.1 Spatial Delayed Alternation

The spatial delayed alternation procedure in the T-maze used in the present experiment has been described by Dudchenko [41] and explored by Maas *et al*. [29] in APO-SUS/ APO-UNSUS rats, with some slight modifications. The maze consisted of 3 arms (50 cm x 14 cm) with a 40 cm wall with a start box of 15 x 14 x 40 cm. At the end of both left-right arms, a food cup (4 x 4 x 4) was placed. In this test, rats had to alternate between the arms to get a reward. On day 1, rats were first habituated for 15 min to the maze with normal food rewards pellets scattered over the maze. On days 2 and 3, one food pellet was placed on both the left and right arms, and rats learned to collect both pellets in less than 3 min within a session of 15 min. On days 4, 5, and 6, the rats received 3, 10, and 30 mg/kg of MAM, 30 min before the test (control groups of SUS and UNSUS received vehicle), and were tested for 10 daily trials. In the first trial of each day, food pellets were placed on both the left and right arms. The rat was placed in the start box, after which the rat was allowed to move freely until a food pellet was retrieved. The rat was placed back in the start box closed by a guillotine door for the 60s of delay and the reward pellet was placed on the opposite side of the chosen one every time. Thus, the rats had to alternate between the arms.

##### 2.5.1.2 Reversal Learning

We used two new naive groups (n = 10 each) of APO-SUS rats. One group was treated acutely with MAM (10 mg/kg), the most effective dose found in the spontaneous delayed alternation test, and the other group was treated with vehicle (control group). On day 1, rats were first habituated for 15 min to the maze with normal food rewards pellets scattered over the maze. The experiment consisted of two phases. During phase 1, only the right arm was blocked by a guillotine, and the animals were forced to visit the left side to catch their food pellets until they ignored completely the right side for 3 consecutive days (days: 2, 3, 4) of 10 trials each. After the first phase, the guillotine was removed and both arms were baited on days 5, 6, and 7. The delay was always a 60s intertrial delay. Thus, the rats were required to adapt their behavior from going to one arm to alternating between two arms. The average of the total correct trials during days 4, 5, and 6 was calculated.

### 2.6 Molecular Exploration of MAM Effects in Post-mortem Rat Brains

#### 2.6.1 RNA Preparation and mRNA Expression Analysis by Quantitative Real-time PCR

Immediately after the gnawing box test, the animals were decapitated and their brains were removed, quickly frozen, and stored at -80^o^C. Brains were coronally sectioned (200 μm) on a Leica cryostat at -12^o^C, and tissue punches were excised from discrete regions of the dorsolateral striatum and nucleus accumbens using a Miltex biopsy puncher (Miltex Inc., York, PA, USA) for both areas (1.5 mm or 1.0 mm diameter respectively), collected in sterile vials, immediately placed on dry ice and stored at −80^o^C until assayed for c-Fos and NPAS4 using RT-PCR. Briefly, total RNA was isolated by a single step of guanidinium isothiocyanate/phenol extraction using PureZol RNA isolation reagent (Bio-Rad Laboratories s.r.l. Italia) according to the manufacturer’s instructions and quantified by spectrophotometric analysis. Following total RNA extraction, the samples were processed for RT-PCR to assess c-Fos and NPAS4 mRNA levels. An aliquot of each sample was treated with DNase to avoid DNA contamination. RNA was analyzed by TaqMan qRT-PCR instrument (CFX384 real-time system, Bio-Rad Laboratories, Italy) using the iScriptTM one-step RT-PCR kit for probes (Bio-Rad Laboratories). Samples were run in 384 well formats in triplicate as multiplexed reactions with a normalizing internal control (Actin). Probe and primer sequences used were purchased from Eurofins MWG‐Operon (Germany) and were as follows: c-Fos forward primer TCCTTACGGACTCCCCAC and reverse primer CTCCGTTTCTCTTCCTCTTCAG; NPAS4 forward primer TCATTGACCCTGCTGACCAT and reverse primer AAGCACCAGTTTGTTGCCTG. Thermal cycling was initiated with incubation at 50°C for 10 min (RNA retrotranscription) and then at 95°C for 5 min (TaqMan polymerase activation). After this initial step, 39 cycles of PCR were performed. Each PCR cycle consisted of heating the samples at 95°C for 10 s to enable the melting process and then for 30 s at 60°C for the annealing and extension reactions. A comparative cycle threshold (Ct) method was used to calculate the relative target gene expression.

#### 2.6.2 Immunohistochemistry

The procedure was adopted from studies by Olivier *et al*. [42] and Nonkes *et al*. [43]. Three weeks following the last PPI behavioural test (when potential immediate effects of stress were expected to be ‘washed out’), rats received either vehicle, CLOZ (10 mg/kg), or MAM (3, 10, 30 mg/kg) subcutaneously, 15 min prior to a subcutaneous injection of DOI (3 mg/kg) or PCP (1.5 mg/kg). Control groups received vehicle + saline. 90 min after DOI or PCP treatment, rats were anesthetized using pentobarbital (60 mg/kg/rat), perfused transcardially with 250 ml of 0.1 mol/l PBS, pH 7.3, followed by 250 ml 4% paraformaldehyde dissolved in 0.1 mol/l of phosphate buffer, pH 7.2 Subsequently, the brains were removed from the skull and post-fixed overnight in 4% paraformaldehyde at 4°C. Before sectioning, the brains were cryoprotected with 30% sucrose in 0.1 mol/l of phosphate buffer for 72 h on a shaker. Brains were fast frozen first on dry ice, and forty-micrometer thick brain sections were cut on a cryostat, and collected in six parallel series in 0.1 mol/l PBS containing 0.1% sodium azide. DAB-nickel-ammonium sulfate method was used to quantify c-Fos protein nucleations in related brain areas.

#### 2.6.3 Image Analysis

For image analysis, we used Image *J* software. The images were converted to grayscale and background areas were taken close to the region of interest. For each region, several measurements were taken for each rat and an average mean gray value and cell counts were determined. All images were cross-checked with Paxinos and Watson’s, The Rat Brain atlas [44], to confirm the presumed location in the brain before measurements were taken. Additionally, all measurements were taken blindly in the treatment groups.

#### 2.6.4 Statistical Analysis

Data are presented as mean ± S.E.M. Sample sizes were chosen using power analysis based on previous studies (with a minimum of 8 rats per treatment group). Randomization and blinding were applied in all experiments. Independent *t-*tests were used for APO-SUS/APO-UNSUS comparisons. One-way ANOVA using Dunnett’s test was used in order to compare different groups with the control group, followed by *post-hoc* Turkey intercomparison test analysis. A two-tailed *P-*value of < 0.05 was considered statistically significant. Statistical analysis was performed using GraphPad version 9 software.

## 3 RESULTS

### 3.1 Structural Analysis of MAM by Proton Nuclear Magnetic Resonance (1H-NMR)

**Fig. (1):**
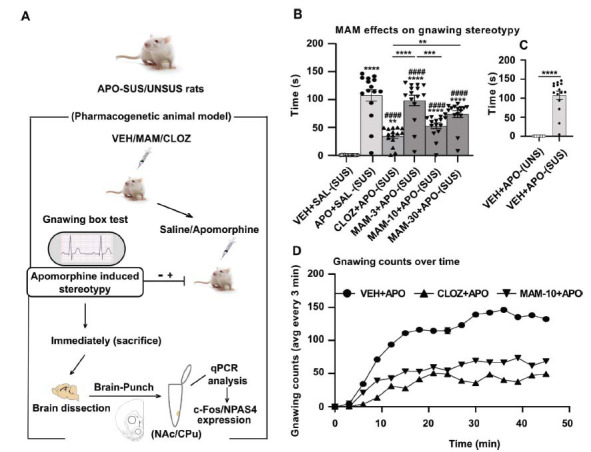
Effects of acute subcutaneous MAM (3, 10, 30 mg/kg) and clozapine (10 mg/kg) pretreatments on the gnawing stereotypy induced by apomorphine (1.5 mg/kg) in APO-SUS rats. (**A**). Schematic representation of the experimental paradigm. (**B**). Gnawing counts in APOSUS rats treated with VEH+SAL (control group), VEH + APO (positive control group), CLOZ (10 mg/kg) + APO (standard group), or MAM (3, 10, 30 mg/kg) + APO. All pretreatments were injected subcutaneously 15 min before APO injection into the neck (n = 9-10 rats per group). (**C**). Gnawing counts in APO-UNSUS compared to APO-SUS rats (independent samples t-test). Values represent average stereotypy gnawing counts every 3 min±SEM. **P < 0.01, ***P < 0.001, ****P < 0.0001 (comparison *vs.* control group/between group’s comparison); ^####^*P* < 0.0001 (comparison *vs.* apomorphine-treated group), using one-way ANOVA, with Dunnett's test, followed by post-hoc Tukey's test. (**D**) Comparative gnawing counts over time during 45 min session in APOSUS rats pre-treated with the most effective dose of MAM (10 mg/kg), CLOZ or VEH, before apomorphine. (A higher resolution/colour version of this figure is available in the electronic copy of the article).

First, we determined the structure of MAM (molecular mass: 181.09; formula: C_9_H_11_NO_3_) using 1H-NMR spectra that exhibited signals for 5 protons (Fig. **1S**). Three singlets at δH 7.47 (1H, dd), 6.85 (1H, dd), and 6.58 (1H, dd) were attributed to the aromatic protons and indicated the presence of a benzene ring. The signals at δH 3.85 (3H, s) and 3.84 (3H, s) were assigned to two methoxy groups. The slightest changes in proton shift (at δH 6.00) have been attributed to the amine group. Complete assignments for all protons were found to agree with the compound structure.

### 3.2 MAM Brain Penetrability

**Fig. (2):**
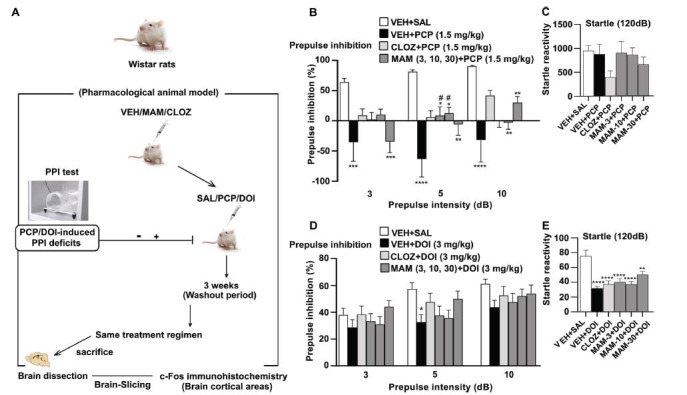
Effects of acute subcutaneous MAM (3, 10, and 30 mg/kg) and clozapine (10 mg/kg) pretreatments on the startle reactivity and prepulse inhibition (PPI) deficits induced by PCP (1.5 mg/kg) or DOI (3 mg/kg) in Wistar rats (**A**). Schematic representation of the experimental paradigm (**C**, **E**). Effects of MAM and CLOZ pretreatment on startle reactivity induced by PCP and DOI (**B**, **D**). Effects of MAM and CLOZ pretreatment on the percentage change of PPI deficits induced by PCP or DOI at different prepulse intensities: 3, 5, and 10dB above the background noise (PP3, PP5, and PP10, respectively). Values represent the average startle in Vmax (**C**, **E**) or average PPI% (**B**, **D**) ± SEM. Oneway ANOVA, with Dunnett's test, followed by post-hoc Tukey's test, was used for comparisons: *P < 0.05, **P < 0.01, ***P < 0.001, ****P < 0.0001 (comparison *vs.* control group); ^#^*P* < 0.05 (comparison *vs*. DOI *vs*. PCP treated group). The male Wistar rats used for both tests are different and naive to both treatments (n = 9-10 rats per group used for PCP and n = 10-11 for DOI). The average PPI (%) ± SEM was calculated as the average responses to all of the pulse-alone or prepulse trials. (A higher resolution/colour version of this figure is available in the electronic copy of the article).

To determine the brain penetrability of MAM, we used high-performance liquid chromatography with the electrochemical detection (HPLC-ECD) method as it is one of the highly sensitive methods for the identification of monoamines. We found that this approach provided reproducible data. Rats were subcutaneously treated with vehicle (blank) or MAM (10 mg/kg). MAM was eluted at 45.32 min and the HPLC-ECD chromatograms of extracts from the three relevant brain areas for antipsychotic activity, the Caudate putamen (CPu), Nucleus accumbens (NAC) and Prelimbic cortex (PrL), showed that MAM reached these three brain regions (Fig. **2S**). These data show that MAM can cross the blood-brain barrier.

### 3.3 Antipsychotic effect of MAM (Antidopaminergic Activity)

#### 3.3.1 Effects of MAM on Stereotypy and Climbing

To assess the antipsychotic activity of MAM, we measured both gnawing stereotypy and hyperlocomotion induced by the D2/D1 receptor agonist apomorphine in MAM-treated APO-SUS rat and Swiss albino mouse models (Fig. **1**). APO-SUS and APO-UNSUS rats were pretreated subcutaneously with the vehicle, 10 mg/kg CLOZ, and 3, 10 and 30 mg/kg MAM 15 min before subcutaneous apomorphine administration at a dose of 1.5 mg/kg. Control groups of APO-SUS and APO-UNSUS rats received vehicle + saline and vehicle + APO (Fig. **1A**). Acute apomorphine administration induced robust gnawing stereotypy in APO-SUS rats compared to APO-UNSUS rats, which did not display any gnawing stereotypy. The independent samples t-test showed a highly significant effect (*t*=10.07, *P* < 0.0001, *df*=28) in APO-SUS *versus* APO-UNSUS rats (Fig. **1C**). The gnawing behaviour induced by apomorphine in APO-SUS rats was antagonized by the D2/5-HT2A-blocking agent CLOZ (10 mg/kg) and by 10 and 30 mg/kg MAM (Fig. **1B**). ANOVA revealed a significant effect of the treatment on stereotypy counts (*F*_(5, 84)_ = 34.57, *P* < 0.0001). Apomorphine treatment of APO-SUS rats (positive control group) led to a robust increase in stereotypy (*P* < 0.0001) compared to those treated with vehicle (negative control group). *Post-hoc* comparisons revealed significant differences in reducing stereotypy by 10 mg/kg MAM (*P <* 0.0001), 30 mg/kg MAM (*P* = 0.0102), and 10 mg/kg CLOZ (*P <* 0.0001) compared to the positive control group. Furthermore, no significant difference was observed between MAM and CLOZ (standard group) at the same dose of 10 mg/kg (Fig. **1B**). The comparative profile of the gnawing counts in APO-SUS pretreated with vehicle, 10 mg/kg MAM or CLOZ, 15 min before apomorphine during the whole session of 45 min is represented in Fig. (**1D**).

**Fig. (3):**
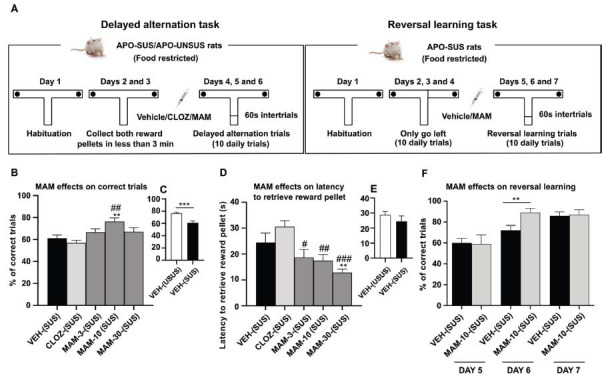
Effects of MAM on spatial working memory deficits in APO-SUS rats (T-maze test) (**A**). Schematic representation of the experimental paradigm (**C**, **E**). Spatial working memory deficit presented in APO-SUS compared to APO-UNSUS rats treated with vehicle and their respective latencies (Independent t-tests, n=8) (**B**, **D**). The effects of vehicle, MAM (3, 10, 30 mg/kg), and CLOZ (10 mg/kg) on spatial working memory deficits presented in APO-SUS rats and their respective latencies (ANOVA, n=8) (**F**). Effect of MAM (10 mg/kg) and vehicle on APO-SUS reversal learning during 3 consecutive days. Values represent the average percentage of correct trials or their latencies to retrieve food reward pellets (±SEM) in 3 daily consecutive trials (30 in total) of the spontaneous delayed alternation. One-way ANOVA, with Dunnett's test, followed by post-hoc Tukey's test was used for comparisons: **P < 0.01, ***P < 0.001 (comparison *vs*. control group), ^#^*P* < 0.05, ^##^*P* < 0.01, ^###^*P* < 0.001 (comparison *vs*. CLOZ treated group).

The mouse climbing test, in which apomorphine induces hyperlocomotion (climbing behaviour), is another popular test for the screening of antipsychotic activity. Three behaviours were taken into account: full climbing (four paws holding the wall), partial climbing (front paws holding the wall), and no climbing (four paws on the floor). Swiss albino mice were pretreated subcutaneously with the vehicle, 2.5 mg/kg of the D2/5-HT2A antagonist, olanzapine or 3 and 10 mg/kg MAM, 15 min before subcutaneous apomorphine administration at a dose of 1 mg/kg. Control groups received vehicle + saline and vehicle + APO. ANOVA revealed a significant main effect of treatment on full climbing time (*F*_(4, 25)_=342.9, *P <* 0.0001) (Fig. **3AS**). Apomorphine treatment of mice (positive control group) led to a robust increase in full climbing (*P* < 0.0001) and remained at or near the top for an average of 18 min compared to the control group treated with vehicle (negative control group). Mice that received pretreatment with 3 mg/kg MAM (*P =* 0.8384) or olanzapine (2.5 mg/kg: *P* = 0.9765) before apomorphine showed no full climbing behaviour. *Post-hoc* comparisons revealed that both 3 mg/kg MAM (*P <* 0.0001) and 2.5 mg/kg olanzapine (*P <* 0.0001) had a significant effect compared to the positive control group. In contrast, 10 mg/kg MAM before apomorphine potentiated full climbing (*P* = 0.0012). No significant difference was observed between olanzapine (standard group) and 3 mg/kg MAM (*P* = 0.9962) in reducing the full climbing (Fig. **3AS**).

However, a distinct profile was observed with 3 mg/kg MAM pretreatment on partial climbing (Fig. **3BS**). While pretreatment with 2.5 mg/kg olanzapine fully antagonized (*P <* 0.001) the full climbing induced by apomorphine, mice pretreated with 3 mg/kg MAM increased partial climbing (Fig. **3BS**). *Post-hoc* comparisons revealed a significant difference (*P* < 0.0001) between the 3 mg/kg MAM pretreated group and the olanzapine standard group in inducing partial climbing.

### 3.4 Atypical Antipsychotic Effects of MAM

To explore the atypicality effects of MAM, we have tested DOI- and PCP-induced PPI deficits in Wistar rats. Male Wistar rats were pretreated subcutaneously with the vehicle, 10 mg/kg CLOZ and 3, 10, 30 mg/kg MAM 15 min before subcutaneous administration of the NMDA receptor antagonist PCP (1.5 mg/kg) or the 5-HT2A/2C receptor agonist DOI (3 mg/kg). Control groups received vehicle + saline (Fig. **2A**). ANOVA revealed a significant main effect of PCP treatment on PPI at all prepulse intensities: PP3 (*F*_(5,53)_=4.349, *P =* 0.0022), PP5 (*F*_(5,53)_=7.289, *P <* 0.0001) and PP10 (*F*_(5, 53)_=5.919, *P =* 0.0002), without affecting the basal startle reactivity (*F*_(5,53)_= 1.504, *P =* 0.2042) (Figs. **2B**, **2C**). In contrast, DOI significantly reduced PPI (Fig. **2D**), but only at PP5 (*F*_(5, 59)_=2.463, *P =* 0.0430), and not at PP3 (*F*_(5, 59)_=1.029, *P =* 0.4101) or PP10 (*F*_(5,59)_=1.020, *P =* 0.4145). Furthermore, DOI affected the basal startle magnitude (*F*_(5,59)_=10.00, *P <* 0.0001) (Fig. **2E**). MAM (3, 10, 30 mg/kg) and CLOZ (10 mg/kg) significantly attenuated the PPI-disruptive effects of PCP and DOI at all doses, which was dependent on the prepulse intensities (Figs. **2B**, **2C**). No significant differences were observed between CLOZ and MAM in the DOI- and PCP-tests.

**Fig. (4):**
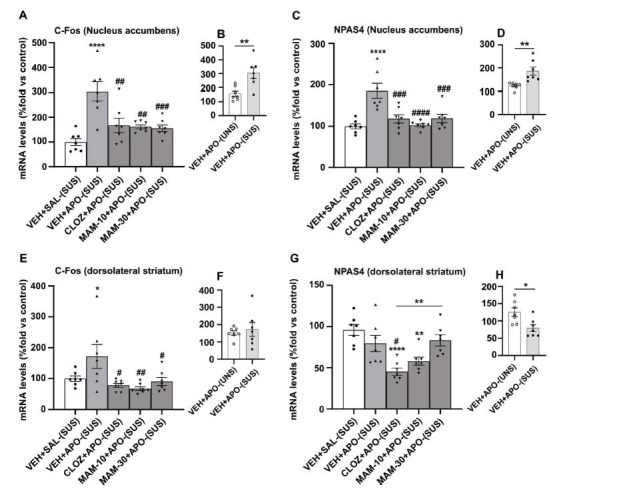
Effects of acute subcutaneous MAM (10, 30 mg/kg) and clozapine (10 mg/kg) pretreatments on c-Fos and NPAS4 mRNA levels induced by apomorphine (1.5 mg/kg) in APO-SUS rats. (**B**, **D**, **F**, **H**). c-Fos and NPAS4 mRNA levels induced by apomorphine in the nucleus accumbens and dorsolateral striatum in APO-UNSUS compared to APO-SUS rats (independent samples t-tests). (**A**, **C**, **E**, **G**). APO-SUS rats treated with VEH+SAL (control group), VEH+APO (positive control group), CLOZ (10 mg/kg)+APO (standard group), or MAM (10, 30 mg/kg)+APO. Values represent the average of %mRNA fold *vs.* control ± SEM. *P < 0.05, **P < 0.01, ****P < 0.0001 (comparison *vs.* control group/between group’s comparison); ^#^*P* < 0.05, ^##^*P* < 0.01, ^###^*P* < 0.001, ^####^*P* < 0.0001 (comparison *vs*. apomorphine-treated group), using one-way ANOVA, with Dunnett's test, followed by post-hoc Tukey's test. All pretreatments were injected subcutaneously 15 min before APO injection into the neck (n = 9-10), and rats were killed immediately after the gnawing box test.

The catalepsy test is another test that is often used as an index of atypicality and predicts possible EPS. Catalepsy is defined as a reduced ability to initiate movement and a failure to achieve a correct posture. TAPs, such as haloperidol, and some of the AAPs (at a higher dose) induce catalepsy in mice, predictive of EPS in humans. Swiss albino mice were subcutaneously treated with 10 or 100 mg/kg MAM, or intraperitoneal haloperidol (1 mg/kg), and ANOVA revealed a significant main effect of treatment on catalepsy time (*F*_(5,30)_=37.54, *P* < 0.0001) (Fig. **4S**). Acute administration of haloperidol induced a significant increase in catalepsy time in mice compared to the control group (*P* < 0.0001). No significant effect on catalepsy was observed with 10 mg/kg (*P* > 0.9999) and 100 mg/kg MAM (*P* > 0.9999) compared to the vehicle control group.

### 3.5 Cognitive Enhancement Effects

#### 3.5.1 Effects of MAM on Spatial Working Memory Deficits in APO-SUS Rats (T-maze Test)

To explore the potential cognitive enhancement effects of MAM against the working memory deficits presented in APO-SUS relative to that of the APO-UNSUS rats, the first step involved examining spatial working memory. The spontaneous and delayed alternation paradigm with a 60-s delay between trials (Fig. **3A**) was used in the first step. Figs. (**3B**, **C**) depicts the effect of acute MAM on the average percentage of correct trials during 3 consecutive daily sessions of 10 trials each (30 trials in total). Independent samples t-test showed a highly significant difference in the time to retrieve a food reward (*t*=4.149, *P* < 0.0001, *df*=46) between APO-SUS *versus* APO-UNSUS rats, but not in the latency to retrieve a food reward (*t*=0.9913, *P* =0.3267, *df*=46) (Figs. **3C**, **E**). The effects of 3, 10, and 30 mg/kg MAM and 10 mg/kg CLOZ on alleviating the spatial working memory deficits (SWMD) presented in APO-SUS rats are also shown in Fig. (**3B**). ANOVA revealed a significant main effect of treatment on total correct trials (F_(4, 115)_ = 5.556, *P =* 0.0004) and a significant main effect of treatment on latency to retrieve food reward (F_(4, 115)_ = 6.635, *P <* 0.0001). Only 10 mg/kg MAM alleviated the SWMD by increasing significantly the total correct trials (*P* < 0.001) in APO-SUS rats compared to the vehicle group. No significant differences were observed between 3 mg/kg MAM (*P =* 0.5255), 30 mg/kg MAM (*P =* 0.4544) and CLOZ (*P =* 0.7475) compared to the control group. *Post-hoc* comparisons revealed significant differences between 10 mg/kg MAM and CLOZ (*P =* 0.0002). Furthermore, MAM decreased dose-dependently the latency to retrieve a reward (Fig. **3D**), but only 30 mg/kg MAM reached a significant effect (*P =* 0.0096) compared to the negative control group. *Post-hoc* comparisons revealed significant differences between CLOZ and 3 mg/kg (*P =* 0.0168), 10 mg/kg (*P =* 0.006), and 30 mg/kg (*P* < 0.0001) MAM in decreasing the latency to retrieve a reward.

#### 3.5.2 Effects of MAM on Reversal Learning in APO-SUS Rats

In the second step, the acute effect of 10 mg/kg MAM, the dose that effectively ameliorated the working memory deficits in APO-SUS in the T-maze test, was compared to that of the vehicle in the reversal learning task (Fig. **3A**). As clozapine did not affect the SWMD, it was not tested in the reversal learning task anymore. The APO-SUS rats were forced to go left on days 2, 3, and 4, until they ignored the right side. Then they were required to switch (reversal) completely from left to right side to get reward pellets on days 5, 6, and 7. Fig. (**3F**) shows the effect of acute 10 mg/kg MAM administration on the average percentage of correct trials during 3 consecutive daily reversal learning sessions (10 trials each). Independent samples t-test showed a significant effect (*t*=2.829, *P* < 0.0111, *df*=18) on day 2 of the reversal in APO-SUS rats acutely treated with MAM *versus* APO-SUS rats treated with vehicle, but not on day 1 (*t*=0.1018, *P*=0.9201, *df*=18) and day 3 (*t*=0.1664, *P* =0.8697, *df*=18).

### 3.6 Brain c-Fos and NPAS4 Expression in Rats Treated with MAM, DOI, or PCP

There are no specific markers available for SZ pathology. Therefore, we assessed the activity of specific brain areas, such as the nucleus accumbens and dorsolateral striatum, which mediate stereotypy and hyperlocomotion and are supposed to be hyperactive in a psychosis-like state. As it is well documented that dopamine agonists mimic the hyperdopaminergic activity [45], we examined whether MAM would be able to reduce apomorphine-induced mRNA expression of the immediate early genes and neuronal activity markers, c-Fos and NPAS4, in these areas. We also assessed the ability of MAM to antagonize neuronal activity induced by DOI [46] and PCP [47] in several cortical areas by counting the number of c-Fos-immunoreactive cells.

#### 3.6.1 Brain c-Fos and NPAS4 mRNA Levels in Rats Treated with MAM

We used RT-PCR to quantify c-Fos and NPAS4 mRNA levels in order to investigate the neuronal activity in the nucleus accumbens and dorsolateral striatum, two brain areas that mediate stereotypy, of APO-UNSUS and APO-SUS rats (Fig. **4**). Apomorphine, the dopamine D2/D1 agonist, is expected to induce hyperactivity in both areas in treated rats.

#### 3.6.2 Nucleus Accumbens

Independent samples t-test showed that apomorphine increased c-Fos (*t*=3.479, *P =* 0.0046, *df*=12) and NPAS4 (NPAS4: *t*=3.477, *P =* 0.0046, *df*=12) mRNA expression levels more strongly in the nucleus accumbens (Figs. **4B**, **D**) of APO-SUS *versus* APO-UNSUS rats.

Apomorphine administration to APO-SUS rats (positive control group), compared to vehicle, led to a robust increase (Figs. **4A**, **C**) in both c-Fos and NPAS4 mRNA levels (*P <* 0.0001). ANOVA revealed a significant main effect of treatment on c-Fos (*F*_(4, 30)_=10.46, *P <* 0.0001) and NPAS4 (*F*_(4, 30)_ =10.96, *P <* 0.0001) mRNA levels. Compared to the positive control group, *post-hoc* comparisons revealed a significant reduction in APO-induced mRNA c-Fos/NPAS4 expression levels after 10 mg/kg (*P =* 0.0014/*P <* 0.0001) and 30 mg/kg (*P =* 0.0009/*P =* 0.0010) MAM as well as 10 mg/kg CLOZ (*P =* 0.0023/*P =* 0.0008). No significant difference was observed between CLOZ (standard group) and 10 and 30 mg/kg MAM.

In summary, c-Fos and NPAS4 mRNA levels induced by apomorphine in APO-SUS rats were alleviated by the pretreatment with CLOZ (10 mg/kg) and MAM (10 and 30 mg/kg).

#### 3.6.3 Dorsolateral Striatum

Independent samples t-test showed that apomorphine significantly decreased mRNA expression levels of NPAS4 (Fig. **4H**) in the dorsolateral striatum (*t*=2.914, *P =* 0.0130, *df*=12) of APO-SUS *versus* APO-UNSUS rats, but did not reach significance with c-Fos (*t*=0.4862, *P =* 0.6356, *df*=12) (Fig. **4F**).

Compared to APO-SUS rats treated with vehicle (control group), ANOVA revealed that apomorphine induced a significant increase in c-Fos mRNA levels (*P =* 0.0419), but there was no effect observed on NPAS4 mRNA levels (*P =* 0.2775). The apomorphine-induced c-Fos increase (Fig. **4E**) was alleviated by pretreatment with both CLOZ and 10 and 30 mg/kg MAM. Compared to the positive control group, *post-hoc* comparisons revealed significant differences in mRNA expression levels with both 10 mg/kg (*P =* 0.0047) and 30 mg/kg MAM (*P =* 0.0406) and CLOZ (*P =* 0.0132). No significant differences were observed between CLOZ (standard group) and 10 and 30 mg/kg MAM.

For NPAS4, a different pattern was found as pretreatment with both CLOZ and 10 mg/kg MAM potentiated a decrease in NPAS4 mRNA expression levels induced by apomorphine (Fig. **4G**). This was revealed by ANOVA in comparison to the control group for 10 mg/kg MAM (0.017), but not for 30 mg/kg MAM (*P =* 0.4949). Compared to the apomorphine-treated APO-SUS group, *post-hoc* comparisons revealed significant differences for the 10 mg/kg (*P =* 0.0047) and 30 mg/kg (*P =* 0.0406) MAM treatment groups as well as the CLOZ (*P =* 0.0132) treatment group. No significant difference was observed between CLOZ (standard group) and 10 mg/kg MAM. Taken together, the effective doses of MAM and CLOZ seem to potentiate a decrease in NPAS4 mRNA expression levels induced by apomorphine in the APO-SUS dorsal striatum.

#### 3.6.4 Brain c-Fos Immunoreactivity in Rats Treated with DOI or PCP

**Fig. (5):**
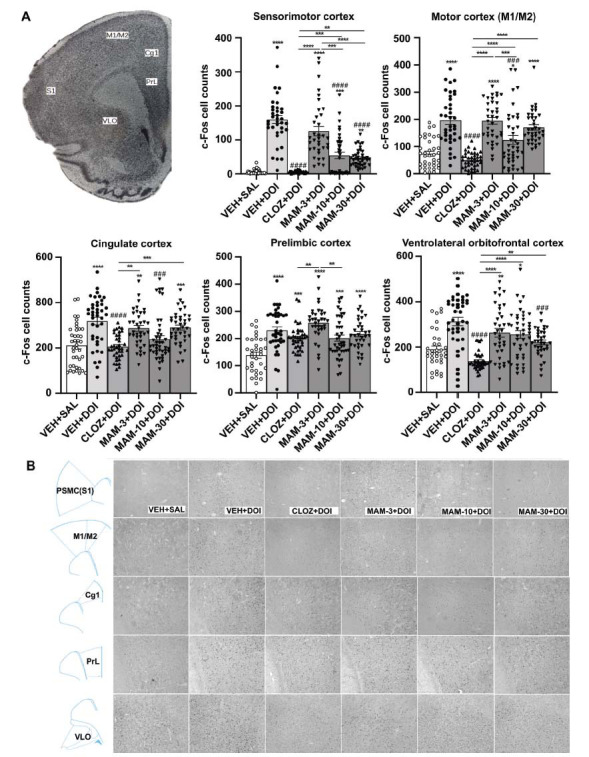
Effects of pretreatment of MAM (3, 10, 30 mg/kg) and clozapine (10 mg/kg) on the c-Fos immunoreactivity induced by DOI (3 mg/kg) in Wistar rats. (**A**) In each area, c-Fos immunoreactive cell counts are present (average ± SEM) in rat cortical areas induced by vehicle+saline or DOI (3 mg/kg) pretreated with vehicle, CLOZ (10 mg/kg), and MAM (3, 10, 30 mg/kg). (**B**) Representative images from each area and treatment. One-way ANOVA, with Dunnett's test, followed by post-hoc Tukey's test, was used for comparisons: *P < 0.05, **P < 0.01, ***P < 0.001, ****P < 0.0001 (comparison *vs.* control group/between group’s comparison), ^###^*P* < 0.001, ^####^*P* < 0.0001 (comparison *vs.* DOI-treated group), in each area. The average number of c-Fos positive cells/region of interest ± SEM (4-5 slices per rat, n=8 rats per group) was calculated using Image J software.

**Fig. (6):**
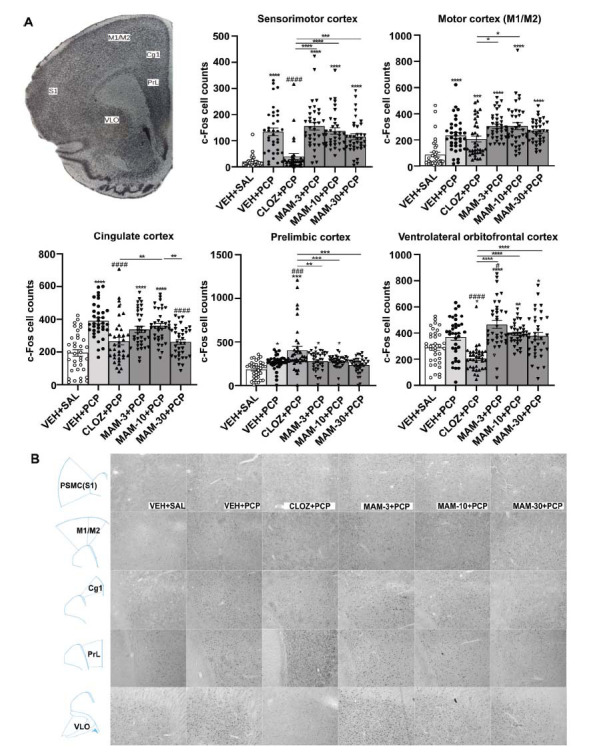
Effects of pretreatment of MAM (3, 10, and 30 mg/kg) and clozapine (10 mg/kg) on the c-Fos immunoreactivity induced by PCP (1.5 mg/kg) in Wistar rats. (**A**) In each area, c-Fos immunoreactive cell counts are present (average ± SEM) in rat cortical areas induced by (VEH+SAL) or PCP (1.5 mg/kg) pretreated with vehicle, CLOZ (10 mg/kg), and MAM (3, 10, 30 mg/kg). (**B**) Representative images from each area and treatment. One-way ANOVA, with Dunnett's test, followed by post-hoc Tukey's test, was used for comparisons: *P < 0.05, **P < 0.01, ***P < 0.001, ****P < 0.0001 (comparison *vs*. control group/between group’s comparison), ^#^*P* < 0.05, ^###^*P* < 0.001, ^####^*P* < 0.0001 (comparison *vs*. CLOZ treated group), in each area. The average number of c-Fos positive cells/region of interest ± SEM (4-5 slices per rat, n=8 rats per group) was calculated using Image J software.

We used c-Fos-immunostaining to examine c-Fos protein expression in SZ-relevant cortical areas of rats treated with DOI (3 mg/kg) or PCP (1.5 mg/kg) (Figs. **5** and **6**). ANOVA revealed a main effect on c-Fos cell counts in response to DOI in many cortical areas, such as the sensorimotor cortex (S1: *F* (5, 226) = 58.60, *P <* 0.0001), the motor cortex (M1/M2: *F* (5, 222) = 15.47, *P <* 0.0001), cingulate cortex (Cg1: *F* (5, 232) = 11.24, *P <* 0.0001), prelimbic cortex (PrL: *F* (5, 218) = 12.70, *P <* 0.0001), and ventral-lateral orbitofrontal cortex (VLO: *F* (5, 221) = 17.28, *P <* 0.0001). Likewise, with PCP, we found significant changes in c-Fos immunoreactivity in the sensorimotor cortex (S1: *F* (5, 201) = 21.79, *P <* 0.0001), the motor cortex (M1/M2: *F* (5, 210) = 15.47, *P <* 0.0001), cingulate cortex (Cg1: *F* (5, 209) = 15.23, *P <* 0.0001), prelimbic cortex (PrL: *F* (5, 216) = 10.90, *P <* 0.0001) and ventral-lateral orbitofrontal cortex (VLO: *F* (5, 208) = 15.14, *P <* 0.0001), respectively. MAM alleviated the strong activation of c-Fos in many cortical areas induced by both DOI and PCP in a dose- and area-specific manner (Figs. **5** and **6**).

### 3.7 Subchronic Hepato-nephrotoxicity of MAM in Swiss Albino Mice

To assess the subchronic toxicity of the most-effective MAM dose found in the present study (10 mg/kg) and 10 times this dose (100 mg/kg), we explored the effects of 28 days of subcutaneous exposure of these doses on blood biochemistry and nephro-hepatopathology in Swiss albino mice.

#### 3.7.1 Body Weight Changes and Histopathology

Subchronic exposure (28 days) of Swiss albino mice to a daily single dose of 10 or 100 mg/kg of MAM did not produce any significant changes in body weights (F_(3,24)_=1.885, *P =* 0.1969) or relative weights of the liver (F_(3,24)_=0.5685, *P =* 0.6411) and kidney (F_(3,24)_ =0.2169, *P =* 0.8837) compared to the control group (Table **1S**). During the 28-day treatment, mice presented normal growth.

The histopathology analysis did not reveal any apparent lesions in the liver and kidney tissue architectures (Fig. **5S**). The examination of the liver tissue illustrated normal cytoarchitecture, with polyhedral hepatocytes arranged radially forming cords around the ventral vein. The kidney showed well-developed renal glomeruli encapsulated with Bowman’s capsule and normal proximal and distal convoluted with tubules.

#### 3.7.2 Biochemical Blood Parameters

Various biochemical blood parameters related to liver and kidney functions were analyzed. ANOVA did not reveal any significant effect on blood levels of the liver- and kidney-related biochemical parameters, including alanine aminotransferase (ALT (*F*_(3,28)_ = 0.3312, *P =* 0.8028)), aspartate aminotransferase (AST (*F*_(3,28)_ = 0.7353, *P =* 0.5398)), total bilirubin (*F*_(3,28)_ = 0.9679, *P =* 0.4217), glucose (*F*_(3,28)_ = 1.322, *P =* 0.2869), total cholesterol (*F*_(3,28)_ = 1.143, *P =* 0.3490), creatinine (*F*_(3,28)_ = 2.042, *P =* 0.1307) and urea (*F*_(3,28)_ = 0.7077, *P =* 0.5555) in 100 mg/kg MAM-treated mice when compared to the control group (Table **2S**).

## 4 DISCUSSION

The results of this study showed a beneficial preclinical effect of MAM against psychosis, without the induction of EPS nor serious toxic effects. Furthermore, MAM did not induce weight gain, hyperglycemia or hyperlipidemia, as presented by AAPs. More importantly, the compound MAM also enhanced cognitive functioning, showing a preclinical profile of a potential atypical antipsychotic drug with major advantages over existing antipsychotic drugs.

The BBB prevents the penetration of 95% of molecules that are tested for drug development, and only very few drug candidates with high lipophilicity and small molecular mass can enter the brain [37, 45]. We found MAM in many brain areas of the injected rodents, such as the prelimbic cortex, nucleus accumbens, and caudate putamen, and thus, the alkaloid crossed the BBB and reached the brain. This finding is not surprising since MAM fits the two criteria of high lipophilicity and small molecular mass.

Toxicity is one of the major challenging aspects of pharmacology research. The liver and kidney are the major targets for xenobiotic toxicity [48]. In this study, the 28-day treatment in mice with 10 times the most effective antipsychotic dose of MAM (100 mg/kg) did not induce any significant differences in body weight, and liver- and kidney-related biochemical blood parameters, nor any gross alterations in both hepatic and nephritic tissues. Therefore, MAM presents a safe toxicity profile at both medium (10 mg/kg) and high (100 mg/kg) dosages without affecting parameters triggered by conventional AAPs, such as weight gain, hyperglycemia, and hyperlipidemia.

To model SZ in rodents, the induction of hyperactivity of dopaminergic brain pathways, primarily midbrain dopamine projections to limbic regions, or the activation of 5-HT2A receptors, induces a state resembling some neurochemical and behavioral features relevant to the positive symptoms (particularly hallucinations) of the disease. These SZ rodent models show hyperlocomotion, sensorimotor gating deficits measured in PPI, and spatial working memory impairments, and these effects are blocked by antipsychotic agents [49]. Since there is no perfect model that reproduces all the complicated symptoms of SZ, we used a combination of these powerful and validated rodent models and behavioural tests for each symptom.

Apomorphine-induced stereotypy and climbing are accepted to be highly characteristic of a psychotic state; thus, these are used for antipsychotic screening activity [50, 51]. An antipsychotic effect is thought to be mediated *via* antidopaminergic activity in certain brain regions, particularly in the mesolimbic system [52]. Most of the currently licensed Aps have been tested and validated using animal models based on dopamine hyperactivity like stereotyped and hyperlocomotion behaviours [17]. The antagonism of apomorphine-induced stereotypy in APO-SUS rats and the climbing in mice by MAM are, therefore, an indication of the potential dopamine-blocking and antipsychotic activity of the alkaloid.

Molecular quantification of c-Fos and NPAS4 mRNA levels in post-mortem brain nucleus accumbens and dorsolateral striatum supported the behavioural results. MAM reduced apomorphine-induced expression of the immediate early genes and neuronal activity markers, c-Fos and NPAS4, in these areas. Together with the fact that MAM exerted an antagonism of apomorphine-induced gnawing in APO-SUS rats, these results predict that MAM exerts antipsychotic-like effects.

PPI, the reduction in the startle response produced by a prepulse stimulus, is diminished in SZ patients [21]. Deficient PPI in SZ patients is a measure of the loss of sensorimotor gating that may represent the interface between psychosis and cognition [20]. Direct serotonin 5-HT2A/2C receptor agonists, such as the hallucinogenic drug DOI, induce PPI-deficits *via* activation of serotonin 5-HT2A and not *via* 5-HT2C receptors [53]. However, DOI-mediated 5-HT2C receptor stimulation could explain the decrease in the basal startle reactivity obtained in pulse-alone trials [39]. In concert with previous reports, the dissociative NMDA antagonist drug PCP induced PPI deficits with all prepulse intensities without affecting the basal startle reactivity. Like DOI, PCP is thought to induce this deficit *via* 5-HT2A receptors [48]. Accordingly, the ability of drugs to reverse both DOI- and PCP-induced disruption of PPI is correlated with their affinity towards serotonin 5-HT2A receptors.

The major finding of the present study was that depending on the prepulse intensities, MAM prevented the PPI-disruptive effects of both DOI and PCP. This is indicative of the atypical effects of MAM since DOI- and PCP-induced PPI disruption in animal models has shown sensitivity to only AAPs [19-21]. The effect of MAM was confirmed by the finding that it reduced the DOI- and PCP-induced increase in c-Fos immunoreactivity in cortical areas.

One of the major side effects of TAPs is the induction of EPS. Haloperidol induces a failure to correct an externally imposed posture in an animal when placed in an unusual posture. This form of catalepsy is observed in “catatonic schizophrenia”, and is important to study as it is strongly regulated by the dopaminergic system [54]. Importantly, MAM did not show any cataleptic effects, even not at the highest dose of 100 mg/kg, suggesting that MAM is devoid of EPS. Catalepsy is also used as an index of atypical activity since only TAPs and some of the AAPs (at a higher dose) induce catalepsy in mice. This effect reinforces the results of the PPI tests regarding MAM atypicality.

Executive functioning, especially working memory and behavioural flexibility, is the most common deficit in SZ [29]. Schizophrenic patients display great difficulty in shifting between different rules or strategies on tests, such as the Wisconsin Card Sorting task. A subset of these patients also displays impairments in reversal learning, a simpler form of behavioral flexibility, entailing shifts between different stimulus-reward associations within a particular dimension [55]. Both spatial discrimination and reversal learning were found to be disrupted in APO-SUS rats. This is not surprising since APO-SUS rats display learning and memory deficits [29, 30]. MAM (10 mg/kg) restored the working memory deficit in both spatial delayed alternation and reversal learning as measured using the T-maze. This is predictive of cognitive-boosting effects. Furthermore, the latency was dose-dependently decreased in APO-SUS rats treated with MAM, which suggests that MAM increases the brain processing speed related to working memory. The results of these two tests are thus predictive of MAM pro-cognitive properties.

Based on our preclinical results, we think that MAM could have at least two main advantages compared to currently commercialized Aps. First, it has the ability to antagonize cognitive symptoms associated with SZ, and second, MAM apparently lacks side effects, especially the major metabolic side effects.

Finally, the molecular receptor binding profile of Aps extends to many neurotransmitter receptors other than the D2/5-HT2A receptors, which makes them the most complicated psychiatric drugs [56]. They may bind to cholinergic (M1, M3), adrenergic (α1, α2, β1), serotonergic (5-HT1A, 5-HT2B, 5-HT2C, 5-HT3, 5-HT7, 5-HT7), dopaminergic (D1, D3/D4), glutamatergic (NMDA channels), glycine transporters, and histaminergic receptors, and even block the serotonin and noradrenaline transporters, and inhibit glycogen synthase kinase-3 [57, 58]. For example, we found that 3 mg/kg MAM induced partial climbing, while different TAPs and AAPs dose-dependently antagonized climbing behaviour [59, 60]. Possibly, MAM exerts partial intrinsic activity at dopamine receptors (likely the D2 receptor), but this is speculative and needs to be addressed in future research. Potentiation of climbing at 10 mg/kg MAM was also observed, comparable to the potentiation of climbing seen with different classes of compounds acting on dopamine D1 receptors [61], α-adrenergic receptors [62], µ-opioids [63, 64] and serotonergic receptors [65, 66]. This may explain the complexity of AAPs by acting on, besides the D2 receptor [67], multiple additional target receptors, and suggests the possibility that MAM could have one (or more) of these targets. Future research may give insight into the receptor-binding profile of MAM and its possible mechanisms of action.

## CONCLUSION

Here, we have demonstrated, for the first time, MAM as an attractive natural “antischizophrenic” drug. Since none of the current Ap medications is able to alleviate the cognitive deficits presented in schizophrenic patients, the observed preclinical cognitive-enhancing effects of MAM make the alkaloid a promising drug for the treatment of cognitive dysfunction in schizophrenia. MAM can cross the blood-brain barrier, thus providing brain bioavailability without inducing weight gain, or altering blood glucose or cholesterol levels.

## LIMITATIONS

Schizophrenia is a complicated psychiatric disorder. Animal models have their limitations and the focus of this study was based on the dopaminergic/serotoninergic theories of schizophrenia using rodent models. Comprehensive multi-level measurements using both sexes of various animal models, *in vivo* microdialysis, and *ex vivo* and *in vitro* receptor binding explorations may further advance the mechanistic understanding of MAM.

## Data Availability

Not applicable.

## LIST OF ABBREVIATIONS

AAPs: Atypical Antipsychotic Drugs
Aps: Antipsychotics
BBB: Blood-brain Barrier
CNS: Central Nervous System
CPu: Caudate Putamen
EPS: Extrapyramidal Symptoms
NAC: Nucleus Accumbens
PPI: Prepulse Inhibition
PrL: Prelimbic Cortex
SZ: Schizophrenia
