# Supplementary material for: The Natural Protoalkaloid Methyl-2-Amino-3-Methoxybenzoate (MAM) Alleviates Positive as well as Cognitive Symptoms in Rat and Mouse Schizophrenia Models
Source: Curr Neuropharmacol. 2023 Jul 24;22(2):323–38. doi: 10.2174/1570159X21666230720122354 (PMC10788887; doi:10.2174/1570159X21666230720122354)
Supplement: Supplementary file 2 — Supplementary material is available on the publisher’s website along with the published article. [file CN-22-323_SD1.pdf]

Supplementary Material

The Natural Protoalkaloid Methyl-2-Amino-3-Methoxybenzoate (MAM) Alleviates Positive as well as Cognitive Symptoms in Rat and Mouse Schizophrenia Models

Yami Bright[1], Dorien A. Maas[1,2,3], Michel M.M. Verheij[1], Maria Serena Paladini[4,5], Helene I.V. Amatdjais-Groenen[6], Raffaella Molteni[7], Marco A. Riva[4,8], Gerard J.M. Martens[2], Judith R. Homberg[1,*]

[1]Department of Cognitive Neuroscience, Donders Institute for Brain, Cognition and Behaviour, Radboud University Medical Centre, Nijmegen, The Netherlands; [2]Department of Molecular Animal Physiology, Donders Institute for Brain, Cognition and Behaviour, Faculty of Science, Nijmegen, The Netherlands; [3]Department of Anatomy and Neurosciences, Amsterdam Neuroscience, Amsterdam UMC, Vrije Universiteit Amsterdam, Amsterdam, The Netherlands; [4]Department of Pharmacological and Biomolecular Sciences, Universita' degli Studi di Milano, Milan, Italy; [5]Altos Labs Bay Area Institute of Science, Altos Labs, Inc., Redwood City, CA, USA; [6]System chemistry, Institute for Molecules and Materials, Radboud University, Nijmegen, The Netherlands; [7]Department of Medical Biotechnology and Translational Medicine, Universita' degli Studi di Milano, Milan, Italy; [8]Biological Psychiatry Unit, IRCCS Istituto Centro San Giovanni di Dio Fatebenefratelli, Brescia, Italy

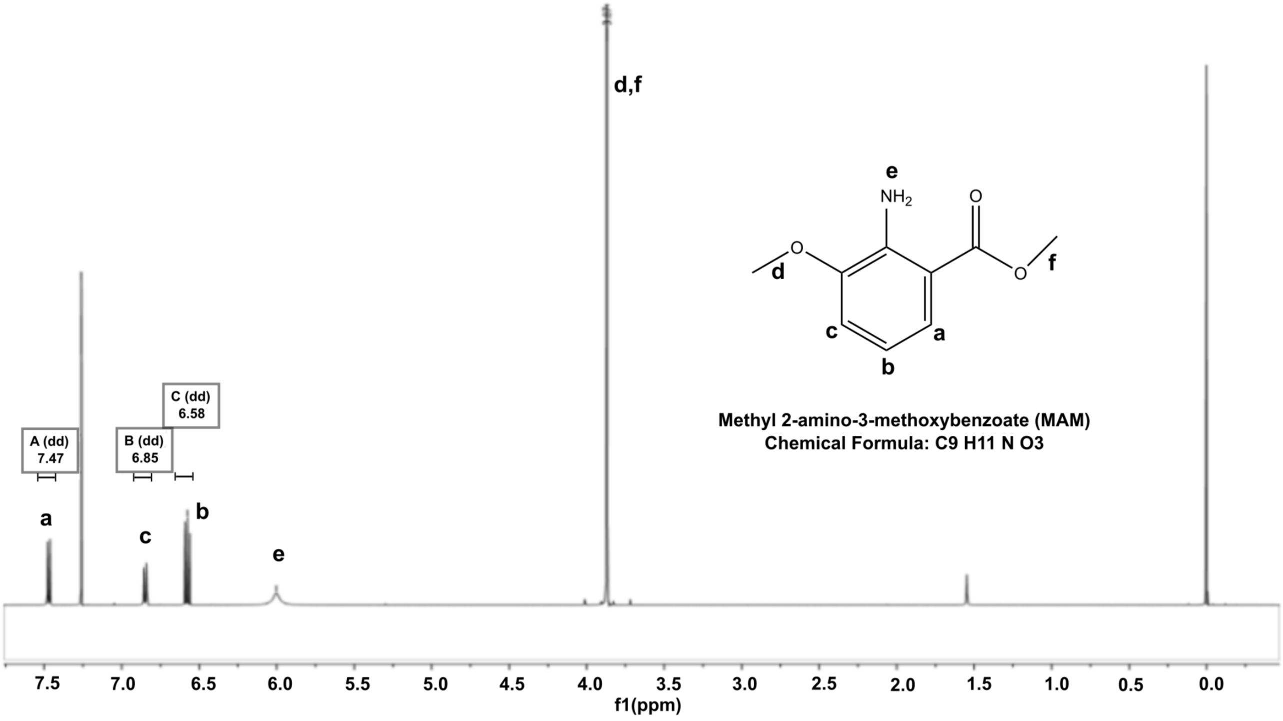

Fig. (1S). NMR spectra for MAM (500 MHz, in $CDCl_3$). ^{1}HNMR (500MHz, CDCl3) 7.47 (dd, J=8.3, 1.3 Hz, 1H), 6.85 (dd, J=7.8, 1.3 Hz, 1H) , 6.58 (dd, J=8.2, 1.3 Hz, 1H).

1875-6190/24 $65.00+.00 © 2024 Bentham Science Publishers

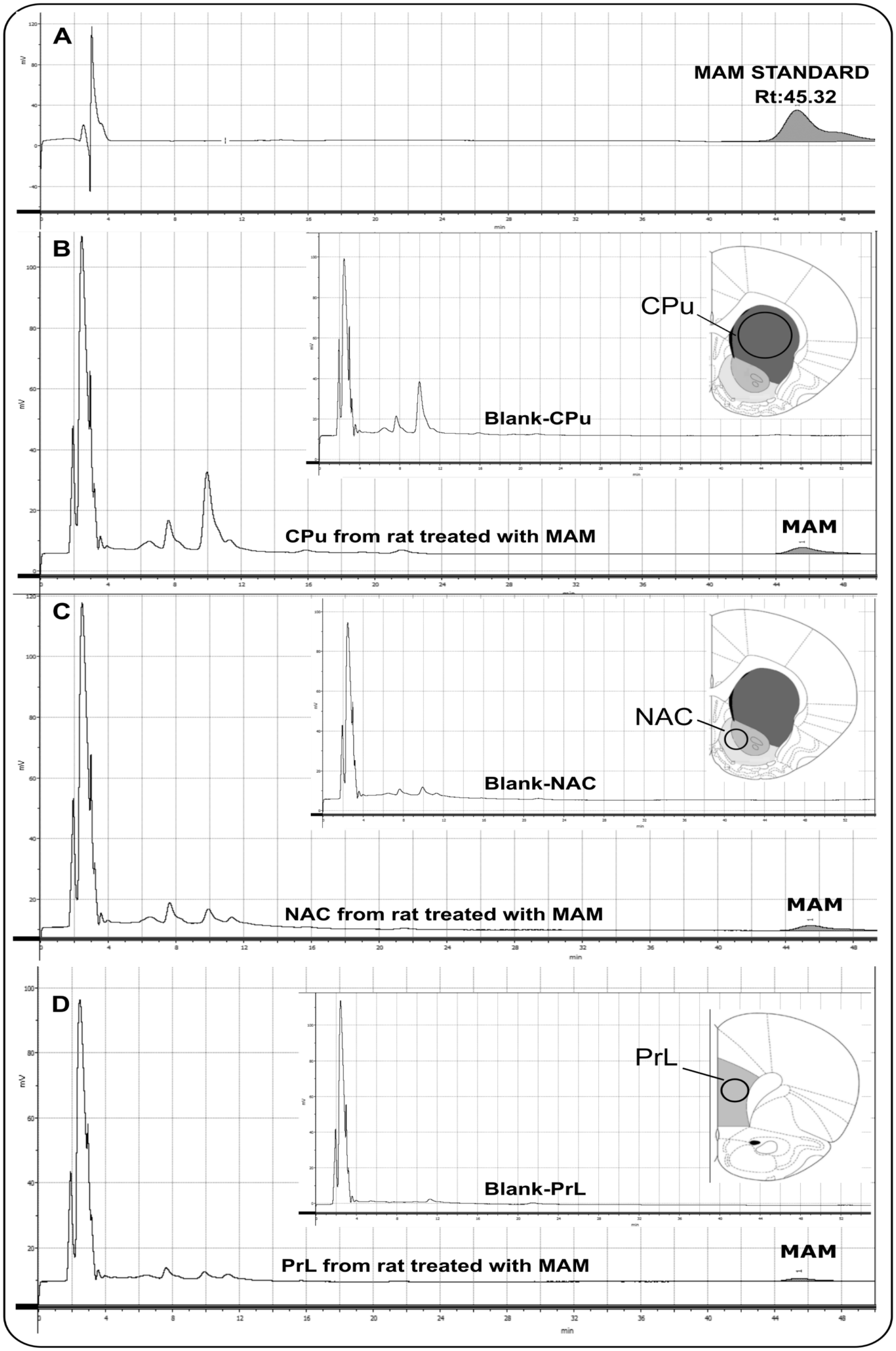

Fig. (2S). HPLC-ECD chromatograms of MAM standard compound and different extracts from brain areas (Caudate Putamen: CPu, Nucleus Accumbens: NAC, and Prelimbic cortex: PrL).

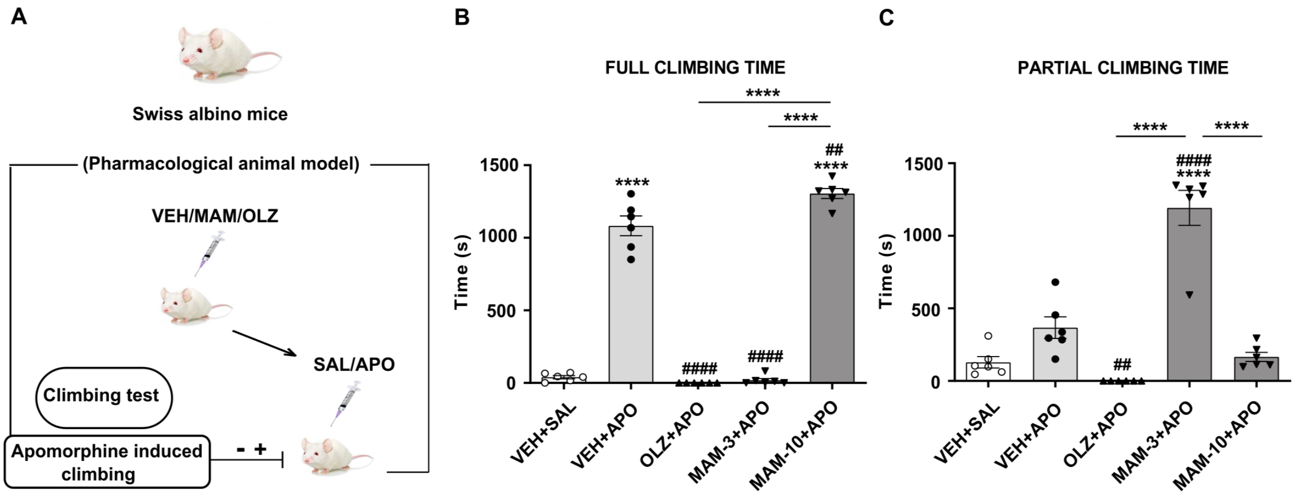

Fig. (3S). Effects of MAM (3, 10 mg/kg) or olanzapine (2.5 mg/kg) on full and partial climbing time in mice. (A). Schematic representation of the experimental paradigm. (B) Full climbing time (four paws holding the wall). **(C)** Partial climbing (fore-feet holding the wall). Data are expressed as mean ± SEM (n=6). Mice were placed first into the cages for 1 h before the experiments to adjust to the new environment, followed by treatment with VEH+SAL (control group), VEH+APO (positive control group), MAM (3, 10 mg/kg)+APO. All pretreatments were injected subcutaneously, 15 min prior to APO (1 mg/kg) subcutaneously into the neck and immediately start testing for a total period of 30min. *P<0.05, **P<0.01, ***P<0.001, ****P<0.0001 (comparison vs control group); #P<0.05, ##P<0.01, ###P<0.001, ####P<0.0001 (comparison vs apomorphine treated group), */ P<0.05 (between group's comparison) using one-way ANOVA, with Dunnett's test, followed by post-hoc Tukey's test.

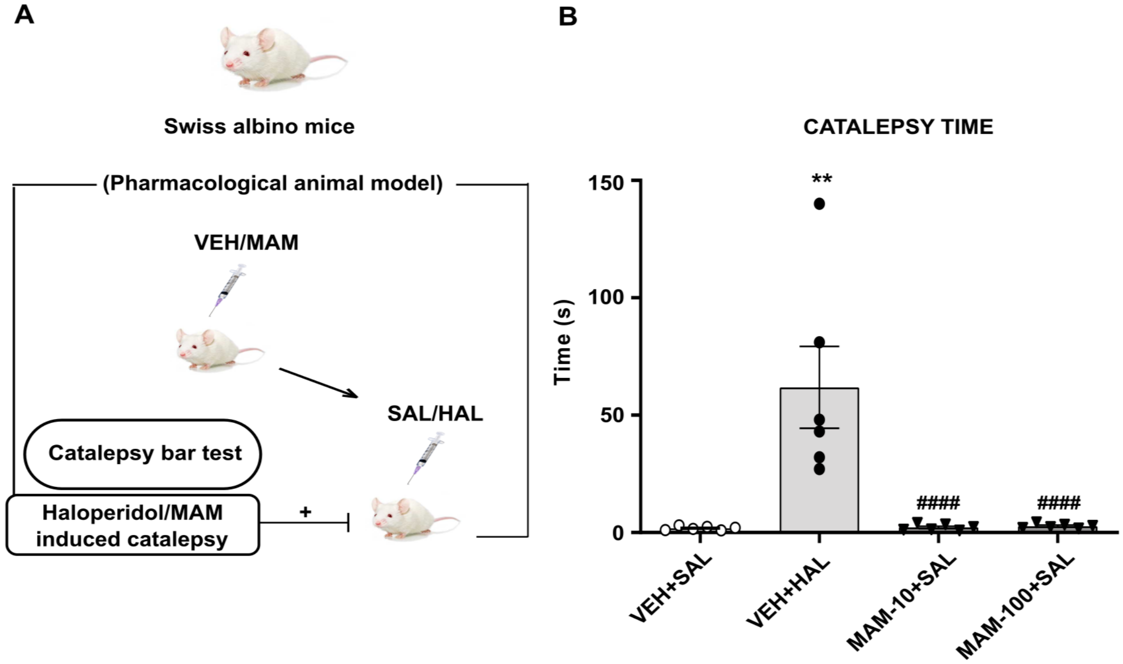

Fig. (4S). Effects of MAM (3, 10 mg/kg) or haloperidol (1 mg/kg) on catalepsy time in mice. Data are expressed as mean ± SEM (n=6). (A). Schematic representation of the experimental paradigm. (B) Mice were treated subcutaneously with VEH+SAL (control group), MAM (10 or 100 mg/kg)+SAL, VEH+HAL (1 mg/kg) and 1h later, mice were subjected to a catalepsy bar test with a maximum cutoff of 180s. *P<0.05, **P<0.01, ***P<0.001, ****P<0.0001 (comparison *vs*. control group); #P<0.05, ##P<0.01, ###P<0.001, ####P<0.0001 (comparison vs haloperidol treated group), */ P<0.05 (between group's comparison) using one-way ANOVA, with Dunnett's test, followed by post-hoc Tukey's test.

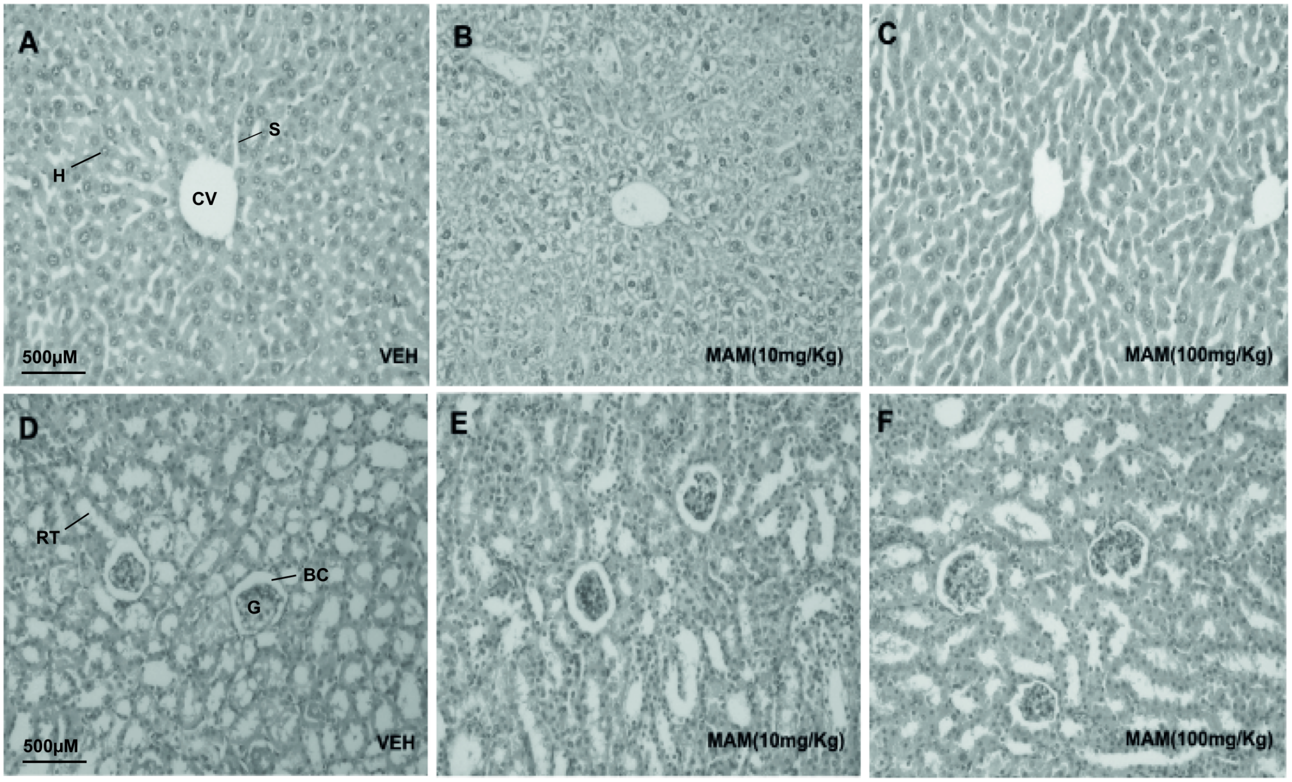

Fig. (5S). Representative liver (up) and kidney (down) tissue sections. Photomicrographs (magnification: X40, hematoxylin and eosin staining) collected from male Swiss Albino mice treated with vehicle (2.5/2.5/95%:DMSO-tween-80-Saline (v:v)) **(A, D)**; MAM (10 mg/kg) **(B, E)**, MAM (100 mg/kg) **(C, F)** during 28 days of treatment. Cental vein (CV); Narrow radiating blood sinusoid (S); polygonal hepatocyte (H); Renal tubule (RT); Glomerulus (G); Bowman's capsule (BC).

Table 1S. Body weight (in grams), relative liver weight (RLW) and relative kidney weight (RKW) after 28 days subcutaneous injection of MAM (10 and 100 mg/kg) to Swiss albino mice.

Parameters	Saline	Vehicle	MAM (10 mg/kg)	MAM (100 mg/kg)
Body weight (day 1)<br>Body weight (day 28)	22.91±0.37<br>30.50±0.45	23.03±0.36<br>31.55±0.52	23.48±0.36<br>32.09±0.56	22.95±0.32<br>31.96±0.66
RLW (%)	4.09±0.15	3.97±0.08	3.91±0.12	4.11±0.13
RKW (%)	1.27±0.03	1.29±0.07	1.23±0.05	1.25±0.05

Values are expressed as mean±SEM (7 mice per group). No significant difference using one way ANOVA, followed by Tukey's multiple comparison test.

Table 2S. Blood biochemical parameters.

Parameters	Saline	Vehicle	MAM (10 mg/kg)	MAM (100 mg/kg)
ALT (IU/dL)	35.85	35.97	37.22	36.76
AST (IU/dL)	43.51	42.44	42.22	41.89
Total bil (mg/dL)	1.08	1.13	0.96	1.08
Urea (mg/dL)	45.46	46.02	44.46	45.43
Creatinine (mg/dL)	0.84	0.87	0.86	0.8
Total chols (mg/dL)	52.96	53.59	53.18	54.93
Glucose (g/L)	1.22	1.13	1.25	1.22

Values are expressed as mean±SEM (7 animals per group). No significant difference using one way ANOVA, followed by Tukey's multiple comparison test.
ALT: Alanine aminotransferase, AST: Aspartate aminotransferase, bil: Bilirubin, chols: Cholesterol.

Supplementary Methods

Chemicals and Reagents

We used analytical-HPLC methanol (RCI labscan, Dublin, Ireland), Analytical-HPLC chloroform from (Merck, Germany), Acetic acid (60.05 g/mol, Sigma-Aldrich, Ireland), perchloric acid (Sigma-Aldrich, Germany), Sodium-1-Octane sulfonate (SOS-high quality, Nacalai tesque, Kyoto, Japan), and EDTA-2NA from Dojindo (Japan). Water was purified using the Millipore Milli-Q Plus system to obtain ultrapure water (18.2 MΩ conductivity) and was used in the preparation of the mobile phase and different solutions.

Structural Analysis of MAM by $_1$H-NMR

To ensure the quality and purity of the purchased MAM before starting its preclinical, both its structure and purity were studied by $_1$H-NMR using a Bruker DMX 500 MHz NMR spectrometer recorded in $CDCl_3$ solution.

MAM Extraction Procedure from Rat Brain

Brains were coronally sectioned (200 μm) on a Leica cryostat at −12° C, and tissue punches were excised from discrete regions of the prelimbic cortex (PrL), caudate-putamen (CPu), and nucleus accumbens (NAC) using a Miltex biopsy puncher (Miltex Inc., York, PA, USA) for CPu (1.5 mm) or PrL and NAC (1.0 mm) diameter respectively, collected in sterile vials, immediately placed on dry ice and stored at −80° C until HPLC analysis. 100 μL of perchloric acid (0.2M) and 3 μL of solution ''A'' were added to each 3 mg of brain tissue in a 2 mL Eppendorf tube and homogenized at a constant speed using a sonicator for 30 s, preincubated for 30 min on ice and then centrifuged (13300 rpm for 15 min) at 4° C. The supernatant obtained was passed through a 0.22 μm PVDF nylon membrane filter (Millipore Millex-GV, Germany) and 20 μL of the collected filtrated supernatant was subjected to HPLC.

Analytical Procedure with HPLC-ECD

The method was applied for the detection of MAM in rat brain tissues. The chromatographic separation of the standards, as well as brain tissue extracts was achieved using a HTEC-510 EICOM PAK (SC-50DS size: 3. Dϕ × 150 mm) over 25° C with ECD detection. The mobile phase was 80% 0.1 M citrate-acetate buffer (made with MilliQ) with 20% methanol including 220 mg/L of sodium sulfonate (SOS) and 5 mg/L of EDTA-2Na.

Antidopaminergic Activity in Mice

Apomorphine Induced Climbing

This test has been described by Costall *et al.* [1]; Peuch *et al.* [2]; Protais *et al.* [3]. Mice that are administered an appropriate dose of apomorphine (a dopamine agonist) will climb the walls of a cage and remain at or near the top for 20-30 minutes (an average of 25 min). Untreated mice on the other hand will occasionally climb up and then climb down rapidly. The exaggerated climbing of apomorphine-treated mice can be antagonized by pretreatment with dopamine blocking agents (atypical antipsychotic, Olanzapine was used as a standard: 2.5 mg/kg). Mice were placed first into cylindrical cages (diameter, 12 cm; height, 14 cm with a wall consisting of metal bars of 0.2 cm diameter; with walls lined with 1 cm_2 wire mesh) surmounted by a wire mesh size 3 mm for 1 h before the experiments to adjust to the new environment. The vehicle itself [DMSO/Tween-80/Saline 2.5% 2.5%/95%: v/v] or MAM (3 and 10 mg/kg) were administered subcutaneously. After 15 minutes, apomorphine HCl was administered subcutaneously into the neck at 1.0 mg/kg, upon which the mice could climb. Mice were scored for different climbing time scores. Three behaviours were taken into account: full climbing (four paws holding the wall), partial climbing (front paws holding the wall) and no climbing (four paws on the floor).

Extrapyramidal Side-effects in Mice

Assessment of Catalepsy

The test was adapted from Sanberg *et al.* [4] and Hoffman and Donovan, [5]. Catalepsy is defined as a reduced ability to initiate movement and a failure to achieve correct posture. Typical Aps such as Haloperidol and some of the atypical Aps (at a higher doses) induce catalepsy in mice, predictive of extrapyramidal side-effects in humans. Mice were handled 5 days before the test. On test day, mice were transferred into individual cages and left undisturbed for at least 1h. Catalepsy was measured by a bar test in which, a 1-cm-diameter metal bar was fixed horizontally 4 cm above the floor. Mice were first pretreated subcutaneously

with saline, 30 min before vehicle [DMSO/Tween-80/Saline 2.5% 2.5%/95%: v/v, s.c], MAM (10 and 100 mg/kg, s.c), or haloperidol (1 mg/kg) and 1 h later, catalepsy was assessed. The forepaws of each mouse were placed gently on the bar and the time in seconds until the mouse took both paws off the bar was recorded, with a maximum cutoff of 180s.

Subchronic Hepato-nephrotoxicity Study

Study Design: A total of 32 adult male Swiss albino mice were used in the sub-chronic hepato-nephrotoxicity study. Mice were randomly divided into 4 groups of 7 mice. Two control groups received respectively normal saline (0.9% NaCl) solution (Group 1) or vehicle [DMSO/Tween-80/Saline 2.5% 2.5%/95%: v/v] (Group 2). Two groups (3 and 4) received respectively 10 mg/kg and 100 mg/kg (dissolved in vehicle) doses of MAM. All treatments were administered subcutaneously in a volume of 5 mL/Kg body weight once daily for 28 days at 10:00 am. The animals were observed for general symptoms of toxicity, external symptoms and mortality. All animals were sacrificed by decapitation under anesthesia 24 h after the last exposure. Blood was collected in heparinized tubes. The criteria of necropsy applied for all animals were based on color changes, size differences, and missing or mislocated organs according to Parkinson et al. [6]. The kidney and liver were removed, cleaned with saline solution, weighed, and preserved in 10% formalin for histopathology examinations. The experiment was conducted according to the protocols described by OECD Guideline 407 [7] with minor modifications. Blood Biochemistry: Plasma was isolated from blood collected in heparinized tubes after centrifugation at 3000 rpm/min for 5 min. Following, biochemical parameters: creatinine, urea, bilirubin, aspartate transaminase (AST), alanine aminotransferase (ALT), glucose, and cholesterol were analyzed using a Beckman automatic analyzer.

Histopathological Study

Fixed liver and kidney tissues were transferred to 70% ethanol. They were then processed using a graded ethanol series (70 to 95%), cleared in xylene, and embedded in paraffin. The paraffin sections were cut into 5 μM-thick slices using a microtome (Leica RM2025 rotary microtome) and stained with hematoxylin and eosin. A Leica (DM2000 LED) light microscope was used to scan the slides for conventional morphological evaluation.

SUPPLEMENTARY REFERENCES

[1] Costall, B.; Naylor, R.J.; Nohria, V. Climbing behaviour induced by apomorphine in mice: A potential model for the detection of neuroleptic activity. *Eur. J. Pharmacol.,* **1978**, *50*(1), 39-50.
http://dx.doi.org/10.1016/0014-2999(78)90251-0 PMID: 28233

[2] Puech, A.J.; Simon, P.; Boissier, J.R. Benzamides and classical neuroleptics: Comparison of their actions using 6 apomorphine induced effects. *Eur. J. Pharmacol.,* **1978**, *50*(4), 291-300.
http://dx.doi.org/10.1016/0014-2999(78)90134-6 PMID: 29758

[3] Protais, P.; Costentin, J.; Schwartz, J.C. Climbing behavior induced by apomorphine in mice: a simple test for the study of dopamine receptors in striatum. *Psychopharmacology (Berl.),* **1976**, *50*(1), 1-6.
http://dx.doi.org/10.1007/BF00634146 PMID: 827755.

[4] Sanberg, P.R.; Bunsey, M.D.; Giordano, M.; Norman, A.B. The catalepsy test: Its ups and downs. *Behav. Neurosci.,* **1988**, *102*(5), 748-759.

[5] Hoffman, D.C.; Donovan, H. Catalepsy as a rodent model for detecting antipsychotic drugs with extrapyramidal side effect liability. *Psychopharmacology (Berl.),* **1995**, *120*(2), 128-133.
http://dx.doi.org/10.1007/BF02246184 PMID: 7480543

[6] Parkinson, C.M.; O'Brien, A.; Albers, T.M.; Simon, M.A.; Clifford, C.B.; Pritchett-Corning, K.R. Diagnostic necropsy and selected tissue and sample collection in rats and mice. *J. Vis. Exp.,* **2011**, (54), e2966. [JoVE].
PMID: 21847084

[7] OECD/OCDE 407. OECD guidelines for the Testing of Chemicals. Repeated Dose 28-day Oral Toxicity Study in Rodents. **2008**.
